# The Effectiveness of Aromatherapy in Reducing Pain: A Systematic Review and Meta-Analysis

**DOI:** 10.1155/2016/8158693

**Published:** 2016-12-14

**Authors:** Shaheen E. Lakhan, Heather Sheafer, Deborah Tepper

**Affiliations:** ^1^Global Neuroscience Initiative Foundation, Los Angeles, CA, USA; ^2^California University of Science and Medicine, School of Medicine, Colton, CA, USA; ^3^Neurological Institute, Cleveland Clinic, Cleveland, OH, USA

## Abstract

*Background.* Aromatherapy refers to the medicinal or therapeutic use of essential oils absorbed through the skin or olfactory system. Recent literature has examined the effectiveness of aromatherapy in treating pain.* Methods.* 12 studies examining the use of aromatherapy for pain management were identified through an electronic database search. A meta-analysis was performed to determine the effects of aromatherapy on pain.* Results.* There is a significant positive effect of aromatherapy (compared to placebo or treatments as usual controls) in reducing pain reported on a visual analog scale (SMD = −1.18, 95% CI: −1.33, −1.03; *p* < 0.0001). Secondary analyses found that aromatherapy is more consistent for treating nociceptive (SMD = −1.57, 95% CI: −1.76, −1.39, *p* < 0.0001) and acute pain (SMD = −1.58, 95% CI: −1.75, −1.40, *p* < 0.0001) than inflammatory (SMD = −0.53, 95% CI: −0.77, −0.29, *p* < 0.0001) and chronic pain (SMD = −0.22, 95% CI: −0.49, 0.05, *p* = 0.001), respectively. Based on the available research, aromatherapy is most effective in treating postoperative pain (SMD = −1.79, 95% CI: −2.08, −1.51, *p* < 0.0001) and obstetrical and gynecological pain (SMD = −1.14, 95% CI: −2.10, −0.19, *p* < 0.0001).* Conclusion.* The findings of this study indicate that aromatherapy can successfully treat pain when combined with conventional treatments.

## 1. Introduction

Aromatherapy refers to the medicinal or therapeutic use of essential oils absorbed through the skin or olfactory system [1, 2]. Essential oils, which are derived from plants, are used to treat illness as well as to enhance physical and psychological well-being. Although the use of distilled plant materials dates back to medieval Persia, the term “aromatherapy” was first used by Rene Maurice Gattefosse in the early 20th century. In his 1937 book,* Aromatherapie*, Gattefosse claimed that herbal medicine could be used to treat virtually any ailment throughout the human organ system. Today, aromatherapy is popular in the United States and around the world [2].

Although many claims have been made relating to the benefits of aromatherapy, most research has focused on its use to manage depression, anxiety, muscle tension, sleep disturbance, nausea, and pain [2]. Some studies suggest that olfactory stimulation related to aromatherapy can result in immediate reduction in pain, as well as changing physiological parameters such as pulse, blood pressure, skin temperature, and brain activity [1]. Although the benefits remain controversial, many patients and healthcare providers are attracted to aromatherapy because of its low cost and minimal side effects. Essential oils currently available for medicinal use are generally recognized as safe by the United States Food and Drug Administration (FDA). In some cases, essential oils can cause minor skin irritation at the site of use. If ingested in large amounts, essential oils can cause phototoxic reactions which can, in rare instances, be lethal [2].

Aromatherapy is most commonly applied topically, or through inhalation. When applied topically, the oil is usually added to carrier oil and used for massage. Essential oils can be inhaled through a humidifier or by soaking gauze and placing it near the patient [2]. Olfactory and tactile sensory stimulation produced by these oils can enhance ordinary human activities such as eating, social interaction, and sexual contact [3]. While more than 40 plant derivatives have been identified for therapeutic use, lavender, eucalyptus, rosemary, chamomile, and peppermint are the most frequently utilized extracts [2].

Even though aromatherapy is commonly used and has been practiced for centuries, few high quality empirical reviews have examined its effectiveness in reducing pain. A database search revealed that common end points for aromatherapy research often focus on the reduction of psychological symptoms such as depression and anxiety or seek to measure the increase of patient satisfaction. Many studies examining the use of aromatherapy in pain reduction focus on therapeutic massage rendering the isolated impact of essential oils without massage unclear. Obstetrical and gynecological pain has garnered the greatest attention when examining the efficacy of aromatherapy. To date, no meta-analysis has expressly examined the use of aromatherapy for pain reduction and management. The aim of this meta-analysis was to quantify the effectiveness of aromatherapy for pain management.

## 2. Methods

### 2.1. Literature Search Strategy

To retrieve available evidence related to the use of aromatherapy for pain management, the author conducted an electronic database search of PubMed, Science Direct, and the Cochrane Library using PRISMA and Cochrane guidelines. Each database was searched using the following MeSH terms:* aromatherapy, essential oils* AND* pain, pain management.* Articles identified in this manner were retrieved and their reference lists searched for additional relevant articles.

### 2.2. Selection of Studies

An initial database search yielded 353 articles related to aromatherapy and pain management. For the qualitative analysis (systematic review), eligible studies were published in English and focused on the use of aromatherapy to manage pain. For the quantitative analysis (meta-analysis), however, several exclusions were used to prevent the analysis of irrelevant or poorly designed studies. Eligible studies were published in English and measured pain on a visual analog scale (VAS). Studies with no pain scale or other measures of pain were excluded. For example, studies that measured the effectiveness of aromatherapy by comparing it to the request or need for an additional pain intervention were excluded. Vague measures such as pleasantness and patient satisfaction were also excluded. Similarly, studies that measured pain and other conditions, such as nausea, in a single scale were excluded. Additionally, studies using measures unrelated to pain such as redness, inflammation, and cardiovascular or respiratory conditions were excluded. Next, only experimental study designs were included. Case studies and studies with no control were excluded. Finally, all eligible studies included at least one measure of pain. Studies that only reported other conditions, such as mood or agitation, were excluded.

### 2.3. Data Extraction

Data was extracted independently for each study included. Although mood measures were included when available, data for nonpain physical measures such as redness, inflammation, and heart rate were not extracted, even when pain was measured. Additionally, data on differences in analgesic use was not extracted. If measures were reported at intervals during treatment, only total mean change or final mean change was used for analysis.

### 2.4. Data Analysis

The primary end point for this study was the use of aromatherapy for pain management. For each study, the standardized mean difference (SMD) of VAS pain between the treatment and control group was calculated. Effect sizes were calculated for all included studies using Stata version 13. Cohen's recommended effect size was considered, with a size of 0.2 indicating a small effect, 0.5 indicating a moderate effect, and 0.8 indicating a large effect. A 95% confidence interval was used to calculate pooled effect sizes reported as standardized mean difference. For studies overall, and each subgroup, heterogeneity was considered high at *I* ^2^ ≥ 75%, moderate at *I* ^2^ = 50%, and low at *I* ^2^ ≤ 25%.

Secondary endpoints included inflammatory pain versus nociceptive pain, chronic versus acute pain, postoperative pain versus nonpostoperative pain, and gynecological pain versus nongynecological pain.

Risk for publication bias was assessed using funnel plots.

## 3. Results

**Figure 1:**
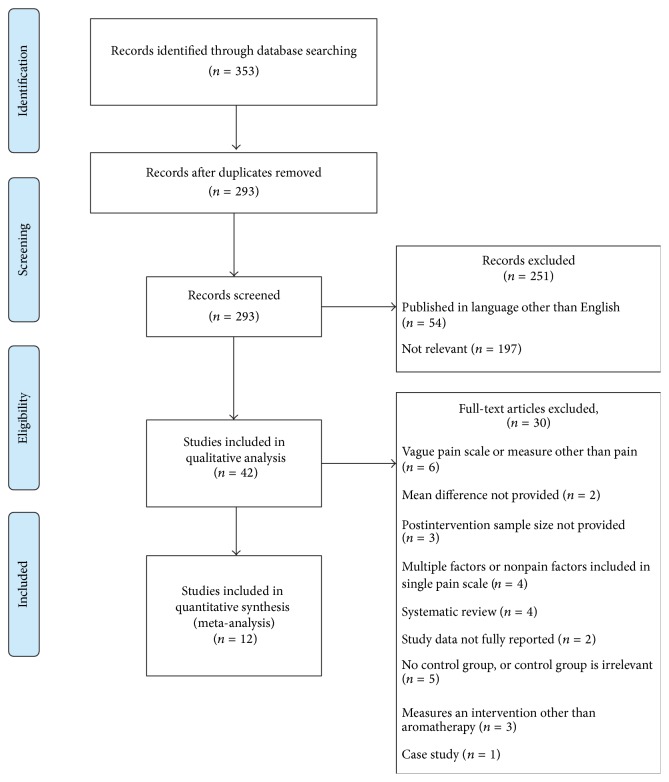
Flowchart of studies that met inclusion/exclusion criteria for qualitative and quantitative analyses.

Of 353 records screened, 42 were included in the qualitative synthesis (systematic review) and 12 in the quantitative synthesis (meta-analysis) (see Figure 1 for flowchart). Those not included in the review were rejected because of poor study design, non-VAS pain measures, reporting conditions not related to pain, or were reviews or meta-analyses (though some of these papers are referenced to provide background information).

### 3.1. Systematic Review

#### 3.1.1. Chronic Pain in Older Adults

As many as 84% of older adults living in nursing homes suffer from chronic pain. Unlike pain among other populations, this chronic pain is persistent, complex, and often not associated with diagnosable conditions. Frequently the pain is associated with stress and poor coping abilities. Chronic pain often leads to other conditions, such as poor sleep, anxiety, depression, and overall reduction in quality of life. A prospective, randomized three-group control trial tested the efficacy of aromatherapy hand massage among nursing home patients suffering from chronic pain. Ailments varied and included physical and psychological complaints such as hypertension, depression, heart disease, arthritis, dementia, healed injuries, and psychiatric illnesses. The majority of patients took daily pain medication and more than half were being treated with antidepressant medication [3].

Participants in the intervention group received hand massage with lavender essential oil while the control group received hand massage alone. A third group had regular nurse visits, but no hand massage. Although both massage groups reported a marked difference in pain and overall well-being, there was no significant difference between the two massage groups. One reason for this finding could be that older adults naturally experience a decreased sense of smell as they age. Sense of smell was not measured at any time during the study, so it is possible that the two massage groups did not experience any difference in treatment [3].

#### 3.1.2. Chronic Back Pain

Approximately 70–85% of older people in the U.S. experience back pain at least once during their lives, with 36% experiencing a period of lower back pain each year [7]. Unspecified lower back pain is among the top 5 most common healthcare provider visits. Treatment can be difficult to get because less than 15% of patients experiencing low back pain are diagnosed with a known cause. Therefore, treatment options tend to focus on symptoms rather than cause [8]. Chronic lower back pain is associated with poor quality of life, reduced physical activities, and often leads to loss of work and productivity. Massage is a common treatment for lower back pain, but the effects of aromatherapy in conjunction with massage are unknown [7].

In a randomized controlled trial to investigate the effect of combining acupressure with lavender essential oil for pain relief of subacute and chronic lower back pain, participants who received a 3-week course of eight sessions of treatment showed a significant reduction in subjective pain intensity and an improvement in objective measures of physical functional performance, including lateral spine flexion and walking time. The results of the study support that acupressure type massage with lavender oil may help improve subacute lower back pain. However there was no group that received acupressure without lavender oil, so it is not possible to say definitively whether the improvement came from the aromatherapy or the massage intervention alone. The researchers recommend that the combined treatment be used along with mainstream medical treatment, as an add-on therapy in reducing lower back pain in the short term [8].

A separate randomized control trial compared participants who received Swedish massage using ginger oil with a control group who received traditional Thai massage through clothes with no oil. In this trial, participants were assessed 15 months after treatment to determine the long-term effects of aromatherapy. The researchers found that both massage groups experienced a significant improvement in pain and mobility. However, the patients whose massage contained ginger oil experienced better outcomes across categories for longer periods of time [7].

#### 3.1.3. Chronic Neck Pain

Like chronic back pain, chronic neck pain can be severely debilitating. An experimental study compared the results of patients who received acupoint electrode stimulation combined with aromatherapy acupressure in addition to conventional treatment versus conventional treatment alone for neck pain. After eight lavender acupressure and acupoint stimulation sessions, the increased intervention group reported an improved range of motion, reduced pain, reduced stiffness, and reduced stress a month after treatment compared to those receiving usual treatment. These results indicate that aromatherapy is a viable option for a complementary treatment in addition to conventional treatment [8].

#### 3.1.4. Chronic Knee Pain

Knee pain is another common form of pain experienced by adults over 50. Chronic knee pain often leads to functional impairment, reducing quality of life. Like other treatments for chronic pain, conventional treatments for knee pain focus on symptoms rather than underlying cause. Many older adults turn to complementary treatment for relief. In a double-blind, placebo-controlled experimental study, massage with ginger oil was compared to a massage only and a treatment as usual group. At one-week follow-up, knee pain and stiffness were similar among the three groups. At the four-week follow-up, the aromatherapy intervention group reported a reduction in knee pain rating. This intervention group also demonstrated an improvement in physical function compared to the control groups. Interestingly, there was no significant change in report of overall quality of life for any of the three groups. Although the results were inconclusive, they suggest that aromatherapy has potential to treat knee pain in addition to standard care [9].

#### 3.1.5. Menstrual Pain

Menstrual pain is extremely common, affecting 25–97% of women worldwide [10]. In about 15% of adolescents and young women, menstrual pain is severe and may impair women from attending work, school, playing sports, or enjoying other activities [11]. In one study, the menstrual pain of women being treated with aromatherapy abdominal massage was compared with a control group of women treated with acetaminophen. The aromatherapy group reported a significantly higher rate of relief than the acetaminophen group. The results, however, are unclear because it is possible that massage alone could alleviate menstrual pain [10]. A later randomized blind placebo clinical trial remediated this by comparing an aromatherapy group with a placebo group, receiving massage with no therapeutic oil. In this study, the aromatherapy group reported a considerable improvement in pain compared to the control [11].

#### 3.1.6. Pain Related to Labor and Childbirth

Despite being a natural process, labor and childbirth is an extremely painful process. Many women are fearful and anxious about the pain, and this anxiety is a common reason for elective cesarean sections. Surgical interventions increase the risk of childbirth complications such as infection, hemorrhage, and thrombosis emboli. Because many women are concerned about the effects of pain medication on themselves and their infants during childbirth, natural deliveries are becoming increasingly more common [12]. In addition to managing pain, aromatherapy during labor and delivery may also decrease nausea, vomiting, headaches, hypertension, and pyrexia [13]. As a result, aromatherapy is becoming a frequently requested nonmedical method of managing pain and promoting relaxation. A further benefit of aromatherapy during labor and delivery is that it decreases the use of medical pain interventions, reducing the cost of care [12]. It is estimated that offering aromatherapy to women in labor would cost approximately $500 per year in a center with 3,000 births per year [13].

Using aromatherapy to manage pain related to childbirth has been researched more than any other specific type of pain. Despite the availability of data, results are inconclusive. A review of two randomized controlled trials involving more than 500 women found no difference in pain intensity, rate of cesarean section, or frequency of requests for pharmacological intervention for women being treated with clary sage, chamomile, lavender, ginger oil, or lemongrass compared to women receiving standard care [14]. A semi-experimental clinical trial found that women who were treated with lavender aromatherapy during labor reported a lower intensity of pain than women in a control group. Unfortunately, the aromatherapy group did not experience a reduced duration of labor or improved Apgar scores of their infants [12]. A similar study using orange oil for pain management during labor and delivery reported comparable results [15]. Although conflicting reports exist, the low cost, ease of use, and noninvasive approach makes aromatherapy a viable option for complementary care during labor and childbirth.

#### 3.1.7. Post-Cesarean Section Pain

Pain is a common complaint after any surgery. Safe and effective pain after cesarean section is very important to the physical and mental well-being of both mother and baby. A single blind clinical trial found that lavender aromatherapy was effective in reducing pain after cesarean section [16]. A triple blind, randomized placebo-controlled trial found the same results and also found that the lavender group reported a 90% satisfaction rate with their treatment, compared to 50% in the placebo group. Although heart rate was the same in both groups, the lavender group experienced less nausea and dizziness than the placebo group [17]. Both studies concluded that although lavender aromatherapy can effectively reduce pain after cesarean section, the serious nature of surgery indicates that aromatherapy should be used as part of a multimodal pain management routine.

#### 3.1.8. Episiotomy Pain

Episiotomy is a common obstetrical procedure around the world, used successfully to prevent lacerations and trauma during vaginal childbirth. A sitz bath is a common treatment recommended by midwives. A clinical trial that compared a conventional sitz bath with use of sitz bath containing lavender found that the lavender treatment did not reduce pain but did reduce inflammation and redness [18]. A separate study, however, found that women who used lavender to manage episiotomy pain used fewer analgesics for pain management during the same time period [19].

#### 3.1.9. Postoperative Pain

Pain is common after almost any surgical procedure. Although analgesic medications are effective in reducing pain and nausea, uncomfortable side effects can prolong the healing process and increase hospitalization time [20]. In a randomized control study to examine pain management after total knee replacement surgery, patients treated with eucalyptus aromatherapy experienced significantly lower pain and blood pressure than the control group [21]. A study that examined lavender aromatherapy in patients recovering from breast biopsy surgery found that the aromatherapy group reported a significantly higher satisfaction with pain management than the control group, even though rates of pain, narcotic use, and discharge time were the same [20].

#### 3.1.10. Hemiplegic Shoulder Pain

As many as 60% of patients who experience complete paralysis of half the body after stroke, a condition known as hemiplegia, complain of shoulder pain. Hemiplegic shoulder pain (HSP) is usually caused by muscle weakness, subluxation, and decreased motor strength. HSP is commonly treated with pharmacological interventions, but the side effects are often unpleasant and dangerous. Nonpharmacological treatments, such as exercise, massage, and biofeedback can reduce pain but are not always effective. A 2007 pilot study examined the benefits of lavender, rosemary, and peppermint oils on relieving HSP. The experimental treatment group received aromatherapy acupressure for 20 minutes twice a day to manage HSP. The pain levels of the treatment group were compared to a control group who received acupressure only without aromatherapy. Although pain was reduced in both groups, the aromatherapy group reported a 30% reduction in pain, compared to 15% reduction in pain for the control group [1].

#### 3.1.11. Pediatric Pain

Treatment of pediatric pain can be complicated. Sedatives and opioids, which are appropriate medications for adults, can impact brain development in young children [22]. Severe pain in pediatric patients is often associated with restricted food and liquid intake, which can cause dehydration [23]. Additionally, many young children are unable to accurately describe their pain to caretakers. Children being treated for serious illness often experience distress not directly related to their illness; therefore a holistic approach to care is an integral part of treatment [22]. In a study that treated infants with lavender aromatherapy for pain associated with blood draw, infants in the aromatherapy group were soothed faster than infants in the control group, even though there was no difference in pain during blood draw [24]. In a study of children recovering from tonsillectomy, children treated with lavender aromatherapy slept better and required 40% less acetaminophen than children in the control group [23]. A study of children who underwent craniofacial surgery, however, found that aromatherapy offered no benefit. The researchers assert that several reasons, including the children being afraid of strangers massaging them, and massage given too soon after general anesthesia may be to blame [22].

#### 3.1.12. Hospice and Cancer Pain

Complementary therapies, such as aromatherapy, are becoming increasingly common in palliative care and cancer treatment units. Nearly three-quarters of UK hospitals offer aromatherapy or massage to hospice and cancer patients. Although few quality studies exist, aromatherapy is believed to reduce pain, anxiety, and depression as well as increase overall sense of well-being. These attributes, in addition to low cost and easy application, make it a viable option for increasing comfort and reducing the use of pain medications [25]. Boehm et al. conducted a meta-analysis of 18 studies examining the effects of aromatherapy on the anxiety, depression, sleep, pain, and overall well-being of cancer patients. Overall, the study concluded that aromatherapy provides short-term benefits to cancer patients. However, many of the studies in the meta-analysis found no significant difference between the aromatherapy and control group. The poor quality of study design, inadequate control interventions, and inconsistent essential oil quality and type created limitations for the study [2]. Similarly, a randomized controlled study involving 17 homecare hospice patients diagnosed with cancer concluded that patients treated with lavender oil and with placebo both reported improved symptoms, compared to the control group. Interestingly, only members of the lavender group chose to continue treatment after the study [25]. A third study was unable to report significant long-term benefits of aromatherapy or massage alone in reducing anxiety or pain. However, this study found statistically significant improvements to sleep scores and depression reduction [26].

#### 3.1.13. Hemodialysis Pain

By the year 2015, nearly three-quarter million Americans will undergo hemodialysis to treat chronic renal failure. Successful treatment requires almost daily needle insertion into a fistula, which creates pain, stress, and anxiety. Pain reduction is necessary to ensure that patients are compliant with treatment. Because of the low cost and ease of administration associated with aromatherapy, it is a viable option for reducing needle insertion pain. A randomized control trial concluded that lavender aromatherapy significantly reduced pain and anxiety in hemodialysis patients [27].

#### 3.1.14. Renal Colic

Renal colic, characterized by severe abdominal and groin pain, is a common condition treated in emergency rooms. Because pain is the presenting problem, narcotics or opiates are often used immediately. In a double-blind, randomized, placebo-controlled interventional studies, patients diagnosed with renal colic were treated with conventional therapy or with conventional therapy combined with rose oil in a vaporizer. The aromatherapy group reported significantly less pain 30 minutes after treatment than the control group [28].

#### 3.1.15. Guillain Barre Syndrome

Guillain Barre Syndrome (GBS) is characterized by sudden paralysis. The paralysis can last as long as 4 weeks before spontaneous recovery begins. Because the paralysis attacks the entire body, a quarter of patients require assisted ventilation. The majority of patients experience a full recovery in 4–6 months. Complications, such as sinus tachycardia, hypotension, and infection, can prolong recovery and in extreme cases lead to death in about 5% of patients [29].

The majority of patients contract GBS following a respiratory or gastrointestinal tract infection. However, GBS can also be triggered by surgeries, HIV, and hepatitis. Fortunately, GBS can be successfully treated. The most common treatment today is intravenous immunoglobulin (IVIG). IVIG treatment is effective in boosting the body's antibody response with minimal complications. A serious drawback to the treatment is that it is painful. GBS patients experiencing facial paralysis are often unable to express their level of distress. Opiate medications are frequently used to manage pain and discomfort during IVIG [29].

#### 3.1.16. Multiple Sclerosis Pain

Multiple sclerosis (MS) is a serious neurological disorder involving myelin loss in the central nervous system and inflammation throughout the body. Symptoms include fatigue, gastrointestinal discomfort, bladder control problems, spasms, and visual disturbances. Three-quarters of MS patients complain of chronic pain. Because MS pain is not relieved easily by conventional methods, many patients believe they must live with it. The discomfort of MS, along with the stress of living with a serious illness, can also cause anxiety and depression [30]. A qualitative study examined the benefits of aromatherapy massage on 50 patients suffering from MS pain. The site of the massage varied based on the pain location of each individual patient, and each patient had a single aromatherapy massage session each month over the course of four months. At the end of the study, most participants said they found it helpful, and 78% chose to continue the therapy. 88% of the patients reported and improved sense of overall well-being, 91% reported improved relaxation, and 55% reported better sleep. Overall, pain medication was reduced by 7% [31]. Although this study provides exciting possibilities for the treatment of MS pain, it is limited by its absence of control group. It is impossible to determine from this study whether the benefit was from the aromatherapy or the massage.

### 3.2. Meta-Analysis

#### 3.2.1. Characteristics of Included Studies

12 studies were included in the meta-analysis. Of these studies, 5 examined inflammatory pain conditions, 7 examined nociceptive pain conditions, 4 studies examined chronic pain conditions, 8 examined acute pain conditions, 3 studies examined postoperative pain, and 6 studies focused on the treatment of obstetrical and gynecological pain.  Table 1 summarizes the methods and findings for each of the included studies, organized alphabetically by authors' last name.

#### 3.2.2. Publication Bias

**Figure 2:**
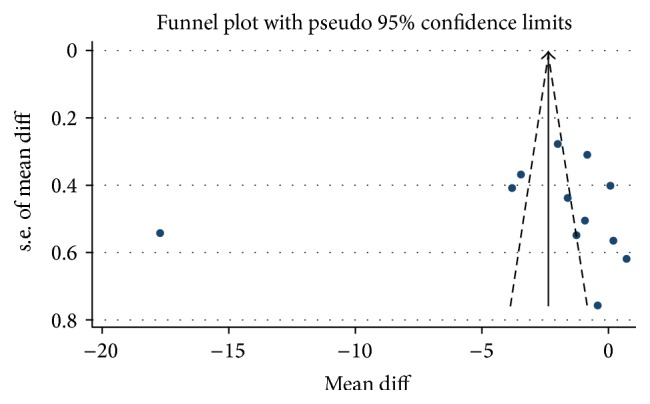
Publication bias funnel plot. A funnel plot was used to assess risk of publication bias. A symmetrical funnel plot is an indicator for lack of bias in a meta-analysis. A funnel plot loses its utility with a cut-off of 10 studies and this analysis included only 12. The funnel plot for this final analysis was not fully symmetrical, but publication bias cannot be concluded based on the small sample size and heterogeneity of studies. The diagonal lines represent the limits of 95% confidence. Because strict 95% limits are not reported, they are referred to as “pseudo 95% confidence limits.”

A funnel plot was used to assess risk of publication bias (see Figure 2). A symmetrical funnel plot is an indicator for lack of bias in a meta-analysis. However there can be many causes for funnel asymmetry including heterogeneity of studies and a small number of included studies. It is said that a funnel plot particularly loses its utility with a cut-off of 10 studies [32], and this analysis included only 12. The funnel plot for this final analysis was not fully symmetrical, but publication bias cannot be concluded based on the small sample size and heterogeneity of studies.

#### 3.2.3. Primary Outcome Measure

**Figure 3:**
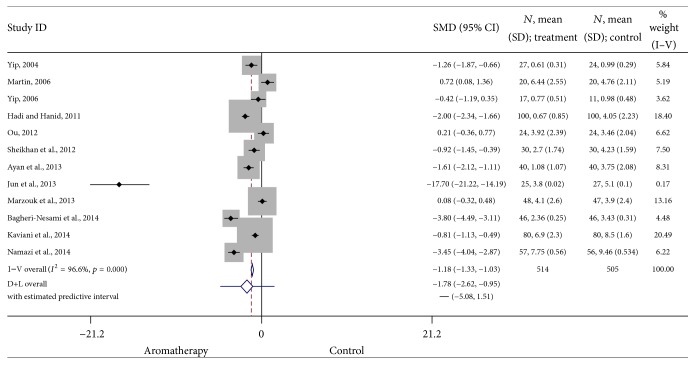
Forest plot: results of all included studies. This forest plot summarizes the results of all included studies. The numbers on the *x*-axis measure treatment effect. The gray squares represent the weight of each study. The larger the sample size, the larger the weight and the size of gray box. The small black boxes with the gray squares represent the point estimate of the effect size and sample size. The black lines on either side of the box represent a 95% confidence interval.

Twelve studies with a total of 1,019 patients were included in the final analysis. The results suggest that the reduction in pain associated with aromatherapy is statistically significant (SMD = −1.18, 95% CI: −1.33, −1.03; *p* < 0.0001). Adhering to Cohen's standards, this indicates a large effect size. Heterogeneity was high (*I* ^2^ = 96.6). The results of these studies are summarized in Figure 3.

#### 3.2.4. Secondary Outcomes Measures

**Figure 4:**
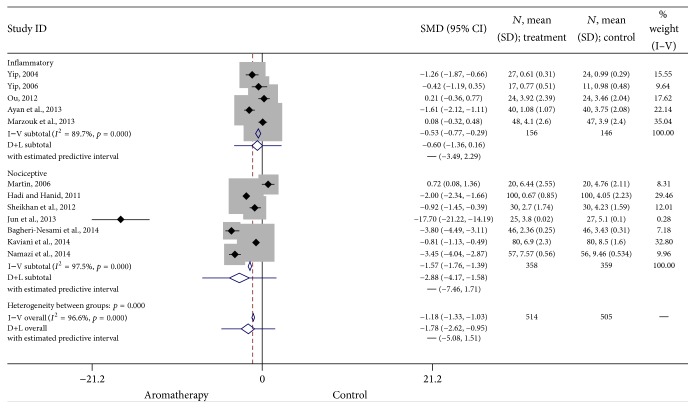
Forest plot: nociceptive versus inflammatory pain. This forest plot summarizes the results of nociceptive pain studies and inflammatory pain studies. The numbers on the *x*-axis measure treatment effect. The gray squares represent the weight of each study. The larger the sample size, the larger the weight and the size of gray box. The small black boxes with the gray squares represent the point estimate of the effect size and sample size. The black lines on either side of the box represent a 95% confidence interval.

*Nociceptive versus Inflammatory Pain.* Five of the eligible studies used aromatherapy to treat inflammatory pain, while seven studies examined nociceptive pain. Subgroup analyses indicated that the efficacy of aromatherapy was more consistent for nociceptive pain (SMD = −1.57, 95% CI: −1.76, −1.39, *p* < 0.0001) than for inflammatory pain (SMD = −0.53, 95% CI: −0.77, −0.29, *p* < 0.0001), although the effect size was large for both. Heterogeneity was high for nociceptive (*I* ^2^ = 97.5) pain and moderately high (*I* ^2^ = 89.7) for inflammatory pain. The results of these studies are summarized in Figure 4.

**Figure 5:**
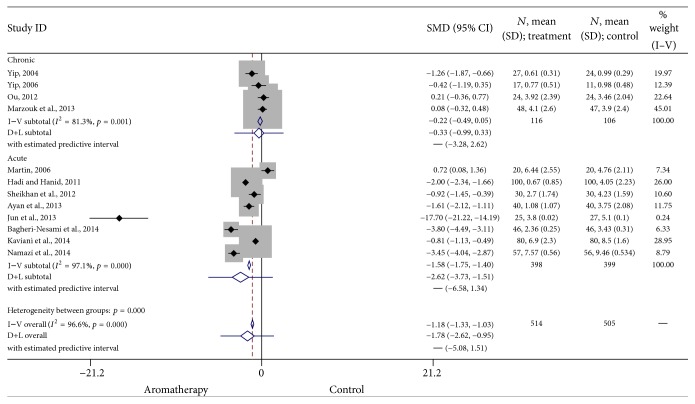
Forest plot: acute versus chronic pain. This forest plot summarizes the results of acute pain studies and chronic pain studies. The numbers on the *x*-axis measure treatment effect. The gray squares represent the weight of each study. The larger the sample size, the larger the weight and the size of gray box. The small black boxes with the gray squares represent the point estimate of the effect size and sample size. The black lines on either side of the box represent a 95% confidence interval.

*Acute Pain versus Chronic Pain.* Four of the included studies examined the use of aromatherapy in treating chronic pain, while eight examined acute pain. Subgroup analyses indicated a large positive effect of aromatherapy on acute pain (SMD = −1.58, 95% CI: −1.75, −1.40, *p* < 0.0001) but only a small positive effect on chronic pain conditions (SMD = −0.22, 95% CI: −0.49, 0.05, *p* = 0.001). Heterogeneity was high for acute (*I* ^2^ = 97.2) pain and moderately high (*I* ^2^ = 81.3) for chronic pain. The results of these studies are summarized in Figure 5.

**Figure 6:**
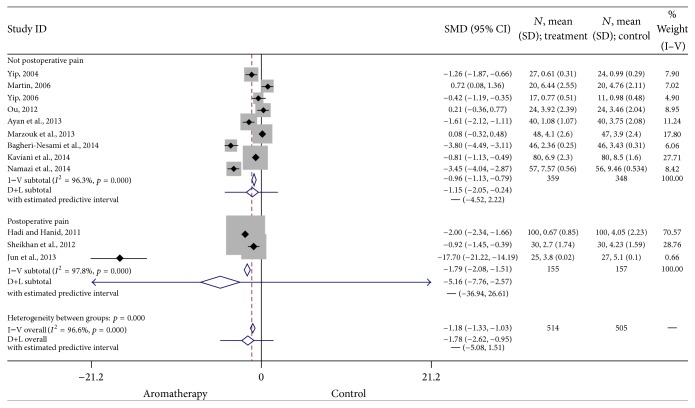
Forest plot: postoperative pain. This forest plot summarizes the results of postoperative pain studies. The numbers on the *x*-axis measure treatment effect. The gray squares represent the weight of each study. The larger the sample size, the larger the weight and the size of gray box. The small black boxes with the gray squares represent the point estimate of the effect size and sample size. The black lines on either side of the box represent a 95% confidence interval.

*Postoperative Pain.* Three studies examined the effect of aromatherapy in managing postoperative pain, while 9 studies focused on pain not related to surgical procedures. Subgroup analyses found a significant positive effect of aromatherapy on postoperative pain (SMD = −1.79, 95% CI: −2.08, −1.51, *p* < 0.0001) and nonpostoperative pain (SMD = −0.96, 95% CI: −1.13, −0.79, *p* < 0.0001). Heterogeneity was high (*I* ^2^ = 97.8). The results of these studies are summarized in Figure 6.

**Figure 7:**
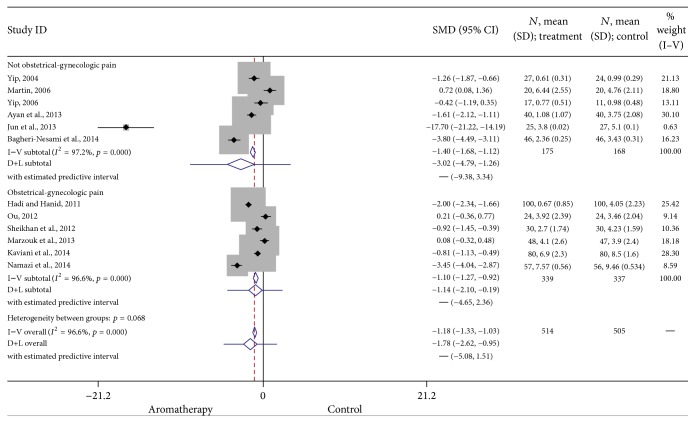
Forest plot: obstetrical and gynecological pain. This forest plot summarizes the results of obstetrical and gynecological pain studies. The numbers on the *x*-axis measure treatment effect. The gray squares represent the weight of each study. The larger the sample size, the larger the weight and the size of gray box. The small black boxes with the gray squares represent the point estimate of the effect size and sample size. The black lines on either side of the box represent a 95% confidence interval.

*Obstetrical and Gynecological Pain.* Aromatherapy is commonly used to manage pain related to menstruation and childbirth. Therefore, these subjects are researched more often than many other types of pain. Six studies included in this analysis examined the benefits of aromatherapy in treating obstetrical and gynecological pain. A significant positive effect was found in these studies (SMD = −1.10, 95% CI: −1.22, −0.92, *p* < 0.0001). Heterogeneity was high (*I* ^2^ = 96.6). The results of these studies are summarized in Figure 7.

## 4. Discussion

Despite being one of the most common complaints of patients in any healthcare setting, pain is extremely subjective and may be difficult for patients to communicate. A holistic approach to pain management takes into consideration the emotional responses, cultural beliefs, cognitive interpretation, and personal history of the patient, in addition to the physiologic aspects of pain [33]. This study found that aromatherapy can be effective in treating pain for a variety of medical conditions. Likewise, most studies found that patient satisfaction was increased, while patient anxiety and depression were decreased. Still, the reasons for these results are unclear. A likely possibility is that satisfaction with pain management often has little correlation to pain reduction and is more often associated with communication, staff behavior, and empathy [33]. This need within the practice of pain management is easily fulfilled by the use of aromatherapy. For one, the touch and attention associated with aromatherapy massage can be beneficial. Massage is typically relaxing and enjoyable for people experiencing many types of pain. In addition to the physical benefits associated with aromatherapy, a pleasant scent may play a key role in patient satisfaction. Most participants who received aromatherapy treatment had the benefit of special treatment sessions outside of normal treatment protocol. The results of this analysis, combined with the findings in the systematic review, indicate that aromatherapy can be beneficial in treating pain when combined with standard pain management protocol. It is also less expensive and has fewer side effects than traditional pain management drugs.

## 5. Study Limitations

The results of this study were impacted by several study limitations. For one, no uniform measure of pain exists. Only studies using VAS were included, which meant that some potentially strong studies needed to be eliminated. Additionally, some studies with robust research designs failed to report pertinent information, such as postintervention sample size or mean difference. Data for other studies was complicated by poor study design, absence of control, or measurements of multiple conditions within a single scale. This study was able to examine the efficacy of aromatherapy for treating nociceptive and inflammatory pain, but no eligible studies examined the efficacy of aromatherapy for treating neuropathic or functional pain. Additionally, the 12 studies included in the final pooled analysis examined treatment of 10 different pain conditions, using varying methods of aromatherapy in the intervention, differing essential oils, and inconsistent control therapies. Because the included studies were conducted in several countries around the world, the cultural attitudes of participants towards aromatherapy must also be considered. Of course, not all patients are equally accepting physical touch. Individual preference, cultural norms, physical illness, or psychological makeup may contribute to touch aversion. Further research is needed to understand the true potential of aromatherapy for pain management.

## 6. Conclusion

This study found a significant positive effect of aromatherapy in reducing pain. These results indicate that aromatherapy should be considered a safe addition to current pain management procedures as no adverse effects were reported in any of the included studies. Additionally, the cost associated with aromatherapy is far less than the cost associated with standard pain management treatment. Although the present meta-analysis indicates a large positive effect for the use of aromatherapy for pain management, the sample size is small. Given the prevalence of aromatherapy, more research is necessary to fully understand clinical applications for its use.

## Figures and Tables

**Table 1:** Studies included in analysis. A summary of the studies included in analysis. CRP = C-reactive protein; VAS = visual analog score; WBC = white blood count.

Study	Study design	Participants (diagnosis, *n*)	Intervention	Comparison	Summary of results
Ayan et al., 2013	Randomized controlled trial, double blind	Renal colic, 80	Rose oil in vaporizer and conventional treatment	Placebo and conventional treatment	There was no statistically significant difference between the starting VAS values of the two groups, but the VAS values 10 or 30 minutes after the initiation of therapy were statistically lower in the group that received conventional therapy plus aromatherapy.

Bagheri-Nesami et al., 2014	Randomized controlled trial	Hemodialysis, 88	Inhaled lavender oil during hemodialysis treatment	Placebo	The mean VAS pain intensity score in the experimental and control groups before the intervention was 3.78 + 0.24 and 4.16 + 0.32, respectively (*p* = 0.35). The mean VAS pain intensity score in the experimental and control groups after three aromatherapy sessions was 2.36 + 0.25 and 3.43 + 0.31, respectively (*p* = 0.009).

Hadi and Hanid, 2011	Clinical trial, single blind	Cesarean section, 200	Lavender oil in face mask with oxygen	Placebo	The aromatherapy group experienced a significant decrease in pain compared to the control group.

Jun et al., 2013	Randomized controlled trial	Postoperative knee replacement, 25	Inhalation of eucalyptus on gauze	Placebo	Pain VAS on all three days (*p* < 0.001) and systolic (*p* < 0.05) and diastolic (*p* = 0.03) blood pressure on the second day were significantly lower in the group inhaling eucalyptus than that inhaling almond oil. Heart rate, CRP, and WBC, however, did not differ significantly in the two groups.

Kaviani et al., 2014	Clinical trial, semi-experimental	Labor pain, 160	Lavender oil on swab attached to patient	Placebo	The mean of pain intensity perception in the aroma group was lower than that of the control group at 30 and 60 minutes after the intervention (*p* < 0.001).

Martin, 2006 [4]	Randomized controlled trial	Hand in ice water, 60	Lemon in oil diffuser	Machine oil in diffuser, no odor	Individuals exposed to both odors reported significantly greater pain than did those in the control condition at 5 minutes. At 15 minutes, individuals exposed to the unpleasant odor experienced greater pain than did the control group.

Marzouk et al., 2013	Randomized controlled trial	Menstrual pain, 95	Abdominal aromatherapy massage	Abdominal massage only	During both treatment phases, the level and duration of menstrual pain and the amount of menstrual bleeding were significantly lower in the aromatherapy group than in the placebo group.

Ou et al., 2012 [5]	Randomized controlled trial, double-blind	Menstrual pain, 48	Self-massage with lavender, clary sage, and marjoram	Placebo	Pain was significantly decreased (*p* < 0.001) after one menstrual cycle intervention in the two groups. The duration of pain was significantly reduced from 2.4 to 1.8 days after aromatherapy intervention in the essential oil group.

Sheikhan et al., 2012	Randomized controlled trial	Episiotomy, 120	Lavender oil in sitz bath on effected area	Treatment as usual	There was a statistical difference in pain intensity scores between the 2 groups after 4 hours (*p* = 0.002), and 5 days (*p* < 0.0001) after episiotomy. However, differences in pain intensity between the two groups, at 12 hours after surgery, were not significant (*p* = 0.066).

Yip et al., 2004	Randomized controlled trial	Low back pain, 51	Acupoint stimulation for relaxation with electrode pads followed by an acupressure massage	Treatment as usual	8 sessions of acupoint stimulation followed by acupressure with aromatic lavender oil were an effective method for short-term low back pain relief.

Yip and Tse, 2006 [6]	Experimental study	Neck pain, 28	Acupressure with lavender oil	Treatment as usual	The baseline VAS for the intervention and control groups were 5.12 and 4.91 out of 10, respectively (*p* = 0.72). One month after the end of treatment, compared to the control group, the manual acupressure group had 23% reduced pain intensity (*p* = 0.02).
